# Sources of Variance in Human Tear Proteomic Samples: Statistical Evaluation, Quality Control, Normalization, and Biological Insight

**DOI:** 10.3390/ijms25031559

**Published:** 2024-01-26

**Authors:** Bella Bruszel, Edit Tóth-Molnár, Tamás Janáky, Zoltán Szabó

**Affiliations:** 1 Department of Medical Chemistry, Albert Szent-Györgyi Medical School, University of Szeged, Dóm tér 8, H-6720 Szeged, Hungary; bruszelbella@gmail.com (B.B.); janaky.tamas@med.u-szeged.hu (T.J.); 2 Department of Ophtalmology, Albert Szent-Györgyi Health Centre, University of Szeged, Korányi Fasor 10-11, H-6720 Szeged, Hungary; toth-molnar.edit@med.u-szeged.hu

**Keywords:** human tears, data independent analysis, quantitative analysis, correlation analysis, normalization, outlier detection, mass spectrometry

## Abstract

Human tear fluid contains numerous compounds, which are present in highly variable amounts owing to the dynamic and multipurpose functions of tears. A better understanding of the level and sources of variance is essential for determining the functions of the different tear components and the limitations of tear samples as a potential biomarker source. In this study, a quantitative proteomic method was used to analyze variations in the tear protein profiles of healthy volunteers. High day-to-day and inter-eye personal variances were observed in the tear volumes, protein content, and composition of the tear samples. Several normalization and outlier exclusion approaches were evaluated to decrease variances. Despite the intrapersonal variances, statistically significant differences and cluster analysis revealed that proteome profile and immunoglobulin composition of tear fluid present personal characteristics. Using correlation analysis, we could identify several correlating protein clusters, mainly related to the source of the proteins. Our study is the first attempt to achieve more insight into the biochemical background of human tears by statistical evaluation of the experimentally observed dynamic behavior of the tear proteome. As a pilot study for determination of personal protein profiles of the tear fluids of individual patients, it contributes to the application of this noninvasively collectible body fluid in personal medicine.

## 1. Introduction

The human tear film is a polyfunctional body fluid whose primary functions are mechanical antimicrobial [1] protection and lubrication [2]. In particular, the tear film was considered to consist of three layers, namely, the outer lipid layer, the aqueous layer, and the inner mucin layer; however, the latter two overlap, and recently have been treated as one continuously varying phase [3]. These layers contain several compound classes, including lipids, small metabolite molecules, peptides, and proteins, in varying concentrations according to their multipurpose dynamic functions. In general, tear proteins are found predominantly in the aqueous layer of the tear film and have a variety of functions. They are secreted from the lacrimal gland and related cells or filtered from the blood. The majority of the proteins secreted from the lacrimal gland are antibacterial proteins (e.g., lysozyme, lipocalin, and lactoferrin); however, proteases and immunoglobulins can also be found in tear fluid [1]. In contrast, plasma proteins (e.g., serum albumin, transferrin, IgG, and IgM) are derived from the blood vessels of the eyelids. As tear fluid proteins originate from different sources, their composition can reflect ocular and systemic diseases [4,5,6,7,8,9,10,11,12]. However, potential environmental factors occurring during or before sample collection can substantially affect the protein composition of the tear fluid. For instance, the normal basal tear differs significantly not only in volume but also in composition from the so-called reflex tear, which is induced by various chemical or mechanical stimuli [13,14,15]. Most tear sample collection methods can induce reflex tear secretion, depending on the subject’s sensitivity, and therefore the collected samples may be variable mixtures of basal and reflex tears containing different layers of the tear film.

Because the sample collection method can affect the highly complex and variable composition of tear fluid, it is a crucial factor for the fluid’s proteomic characterization. Two methods commonly reported in the literature are the Schirmer strip method, where an absorbent is placed into direct contact with the eye, and the glass capillary method [3,16]. The latter can be combined with flushing of the eye surface with a saline solution [13]. Nättinen et al. [17] have conducted a quantitative proteomic comparison of samples collected using a capillary or the Schirmer strip method. They have identified a higher number of proteins and observed enrichment of intracellular proteins in Schirmer strip samples, while immune response-related extracellular proteins were found to be enriched in capillary samples.

Several comprehensive studies on the protein composition of tears have recently been published, enabled by advancements in analytical instrumentation. Using a one-dimensional separation process, 60–309 proteins were identified [18,19]. This number was increased to 491–1543 by application of multidimensional liquid chromatography–mass spectrometry [19,20]. In 2013, The Human Eye Proteome Project (EyeOME) was started in order to build a comprehensive protein database of all proteins identified from eye tissues and fluids [21]. This database currently contains 1509 proteins identified from human tear fluid. However, none of the above studies investigated the quantitative aspects of the tear protein profile. Moreover, possible day-to-day differences and intra- and interpersonal variances in tear composition were not examined, since pooled samples were used for the qualitative mapping of the human tear proteome.

In the literature, only a small number of studies have evaluated intrapersonal (day-to-day and left eye–right eye) variances in the protein composition of human tears, and these have focused either on immunoassay-based quantitation of a limited set of selected proteins [20,22,23,24] or on variations in the “fingerprints” of low-mass, intact proteins by matrix-assisted laser desorption/ionization time-of-flight spectrometry [25]. Dammeier et al. [26] recently quantified several metabolites and proteins in tear samples from both eyes of 12 individuals. Samples were collected at three random time points using Schirmer strips. Their conclusions on the proteomic variance between eyes and with time are limited to the 15 selected proteins and represent information on Schirmer strips. Recently Ponzini et al. performed time-dependent analysis using a deeper proteomic approach; however, that was not the main focus of their work, and it is limited to one eye of two individuals and three timepoints [27]. Lepine et al. [28] recently performed targeted LC–MS analysis of 226 proteins in repeatedly collected tear samples. The results and conclusions in this work were based on Schirmer’s strip samples collected on three different days. They concluded that variance caused by proteoforms cannot be excluded, as their targeted quantitation was performed using a single peptide of each protein; on the other hand, the contribution of technical variance of the protocol was not determined. Besides these limitations, no efforts were made to decrease variance, and no intrapersonal eye-to-eye comparisons were performed.

The present study was designed to provide preliminary data on normal daily variations and eye-to-eye differences in the protein profiles of human tear fluid samples collected by capillary. The biological variations are, however, not easily distinguished from effects caused by sample collection, processing, and data analysis. Sample collection may cause different levels of changes in tear composition due to induction of reflex tearing depending on sampling conditions and individual sensitivity [3]. Sample processing adds some level of technical variance which can be estimated from replicate samples. Generally, during quantitative proteomics analysis, the possible presence of different proteoforms is neglected, although genetical variants, post-translational modifications, alternative splicing, or proteolysis may cause differences in detected peptide quantities. Analysis of the extent of these effects on the detected proteome composition may help to evaluate the biological/medical relevance of analysis of single tear samples [27], expanding the current knowledge on the significant effect of tear sample collection on protein composition [29]. The analysis provides biologically relevant information on the variations of individual proteins and may lead to the identification of correlated protein clusters in human tears. This information is highly valuable in the identification of tear specific source, function, and localization (e.g., layers of tear fluid) of proteins in this complex body fluid. Such body fluid-specific protein annotations are not available in current protein databases, hindering the meaningful biostatistical evaluation (e.g., enrichment analysis) of quantitative proteomics data. These correlational analyses also result in important insights on the level and sources of variance that may support further development of tear biomarkers and their application in precision medicine [30,31]. Our conclusions on personal sample-to-sample correlations may also serve as guidelines for quality control, normalization, and exclusion of non-representative tear samples. We also evaluate the possibility of statistical comparison of the tear proteome profiles of healthy individuals based on repeated sampling, which may serve the aims of precision medicine.

## 2. Results and Discussion

### 2.1. Ophthalmological Examination

Three volunteers (D1, D2, and D3) participated in this study. A detailed description of the participants’ demographics and the results of the ocular surface examinations can be found in Table 1. Ophthalmological examinations did not reveal any pathological abnormalities. The tear film breakup time and the Schirmer I test results were within the normal limits. In addition, no fluorescent punctate staining could be detected on the ocular surface, and the eyelids and meibomian glands were physiologically normal.

### 2.2. Global Parameters of Tear Samples

Total protein contents and tear volumes of collected tear samples were measured, right after the 2 min long sample collection. The day-to-day variance was evaluated for both eyes (OD: oculus dexter/right eye, OS: oculus sinister/left eye) of each donor. The level of daily variation of tear volume, protein concentrations, and the difference in these parameters between the two eyes are shown using bar plot diagrams in Supplementary Figure S2. The relation between total protein concentrations and collected tear volume was also investigated, but they did not show significant correlation (Pearson r = 0.234, *p* = 0.21).

Intra- and interpersonal variances in the tear volumes and protein concentrations were also examined (Supplementary Table S1). In spite of the controlled sample collection protocol, high interpersonal and intrapersonal variances were found in the tear volumes (overall CV: 71%), whereas the overall variance in the protein concentration was around 35%, which is consistent with other studies [23].

Notable variations were observed in the left eye/right eye volume ratios, while a lower variance was observed for the protein concentration ratios (i.e., the ratios were close to one) (Supplementary Figure S2). For one subject a significant difference was found in measured tear volume between the two eyes (D1, *p* < 0.05 in paired Welch’s *t*-test) in our capillary collection method, while there was no difference in the ophthalmological Schirmer’s test. This difference may be due to the balancing effect of the stronger stimulus caused by the Schirmer’s strip [3].

### 2.3. Proteomics Results

#### 2.3.1. Composition of the Identified Tear Proteome

A spectral library was built for the quantitation of proteins detected from the DDA LC–MS analysis of the pooled samples, including 19 1DGE gel slices and three individual liquid pools (see the details in Section 3.4). A total of 455 distinguishable protein groups were identified. For a complete list of identified proteins, see Supplementary Table S2.

In order to compare our protein library to protein identifications currently available in the literature, the EyeOME [21] was identified as the most comprehensive source of data. The human tear-specific section of the EyeOME project contains 1509 proteins and includes all proteins identified in our work, with the exception of immunoglobulins, which are excluded from the EyeOME project. However, it should be noted that the majority of the proteins in the EyeOME database originated from studies using multidimensional LC–MS analysis of tear samples collected with Schirmer strips [18,19,32]. Our study resulted in a similar number of protein identifications as other one-dimensional LC–MS studies [18], while it was limited to tear fluid proteins as a consequence of the capillary sample collection method.

The top 30 proteins represent 97% of the total protein amount, and their summed intensity covered two orders of magnitude, while common proteins present in all samples give more than 99%. The top 30 proteins, except for keratins, are all secreted, and more than 70% are involved in responses to chemical or immune stimuli according to the assigned Gene Ontology terms. Among the identified proteins, the largest protein classes were different immunoglobulin chains (Ig, 72), keratins (14), and hydrolases (22). According to Uniprot annotations, 40% of the identified proteins are glycoproteins, and 39% are phosphorylated.

For the determination of protein level variances, protein–protein correlation, and differential analysis, a spectral library was created using standard assumptions for quantitative analysis: fully tryptic peptides with a maximum of two missed cleavage sites, and methionine oxidation as variable modification. In total, 2936 tryptic peptides were identified this way. Peptides with post-translational modifications and non-tryptic cleavage sites were identified in additional steps. In order to limit database search space and false discovery rate, these identifications were performed separately using a sequence database of proteins already identified. A total of 1308 semitryptic and 184 modified peptides were added this way to the library. Sequence alignment of these peptide forms showed that 56% of the tryptic peptides had overlapping regions with identified semitryptic or missed-cleavage peptides, while 11% with modified peptides; thus, protein quantification may be affected by these.

In the DIA measurements, a total of 331 protein groups were quantified based on the combined DDA spectral library. While 199 protein groups were common in 90% of all individual samples, covering almost five orders of magnitude in summed MS intensity, 275 proteins were detected in at least three samples of each person.

#### 2.3.2. Quantitative Variance in the Composition of the Human Tear Proteome

Several sources can contribute to the variance observed in proteomics analysis of human tears: (1) internal (e.g., health) or external changes in subjects’ biological state before and at the time of sampling but unaffected by sample collection; (2) sampling variance caused by stimulus, injury, etc., occurring during sample collection; and (3) technical variance caused by uncontrolled parameters of sample processing and analysis. In addition to those effects, which alter protein concentrations, variations in individual tryptic peptides used for protein quantification can cause pseudo changes in measured protein abundance. Most commonly, post-translational modifications with relatively high site occupancy and protein cleavage by enzymes with different specificity than trypsin can cause such variations. As a large fraction of known tear proteins are extensively modified and tear fluid contains several proteases, these factors should also be considered. In the following analysis, we make efforts to differentiate and determine the respective contributions of those sources of variance.

The quantitative reproducibility of our digestion protocol and the level of technical variance were first determined. To this end, the digestion and MS/MS analysis of a randomly selected sample were repeated in triplicate. The median of the technical variance for all quantified proteins was found to be 14%, whereas 74% of the proteins had <20% and 62% of the proteins had <10% technical CV (see Supplementary Figure S3 for total technical variance of precursor ions and proteins as a function of their average intensity).

**Figure 1:**
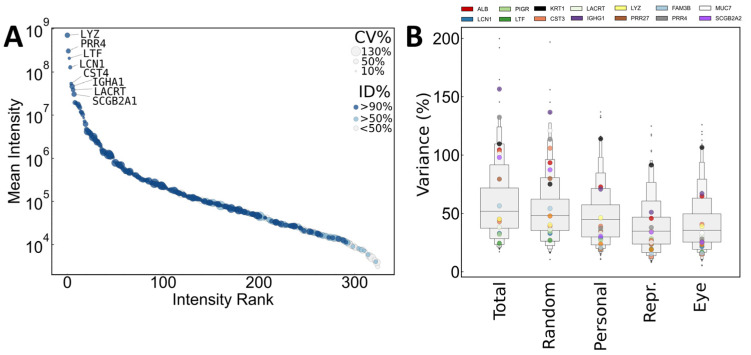
(**A**) Abundance distribution of quantified proteins shown as mean intensity of all (*n* = 30) samples. Dots are sized proportional to observed intrapersonal variance. Dots are colored according to identification frequency as a percentage of all samples. Proteins with mean intensity higher than 5% of highest abundance protein are labeled with gene names. (**B**) Letterplot representations of total intra-/interpersonal and intra-eye variance distributions of normalized protein intensities (MaxLFQ). % CV distributions of all quantified proteins are shown as medians of all eyes/persons/random groups. Values for selected major tear proteins are shown with colored dots. Interpersonal variance is estimated based on randomized groups of the same size (*n* = 10 in each group). Intrapersonal variance of representative (Repr.) samples are calculated from the representative samples (*n* = 7 for each person) selected by the method described in Section 3.2. Intensities were normalized to the sum of protein intensities.

To compare interpersonal and intrapersonal variance based on the same sample size (*n* = 10 in each group), variances were also calculated in randomized groups containing a similar number of random samples from each person. Relatively high variance was observed in protein quantities, even in the case of the most abundant proteins (Figure 1). Only keratins were found to have a common extraordinarily high variance in tear samples of all persons.

Currently, limited information is available in the literature on tear proteome variance. While the immunochemical measurements [13,20,22,24] and LC–MS-based targeted analysis [26,33] of selected proteins showed similar levels of interpersonal or intrapersonal variations as our measurements, no efforts were made to differentiate sampling and biological variations. The most comprehensive study on this topic was performed by Lépine et al. [28] recently, following variations of 226 proteins in tear samples collected by Schirmer’s strip for three days. We have found a significant correlation (Pearson r = 0.55, *p* < 10^−5^) between intrapersonal variance in our data and inter-day variance in that paper based on commonly quantified proteins, despite the difference in sample collection methods. Supplementary Figure S4 shows the similarity in intrapersonal and interpersonal CV % distributions of the two data sets, when all samples were involved, and the same normalization approach (summed intensity) was applied. In Section 3.2, we present some methods to successfully reduce sample-to-sample variances.

Quantitative proteomics data and annotations for all the quantified proteins can be found in Supplementary Table S3.

To put our data into context, the inter- and intrapersonal variances of the human tear fluid observed in this study should be compared with the variances of other body fluids that are commonly used as biomarker sources, including human plasma and saliva. P. E. Geyer and his colleague determined the intra- and interpersonal variances of human plasma [34]. They found that the human plasma proteome is stable over time and has greater inter- than intrapersonal variability (19% and 5.5% of the quantified proteins showed less than 20% inter- and intrapersonal CV, respectively). In contrast, in the human tear proteome, 4.63% of the quantified proteins showed less than 20 CV% intrapersonal variance, and 0.4% of the quantified proteins showed less than 20 CV% interpersonal variance. These results indicate that the human tear proteome changes more dynamically over time, and this variance is comparable to the interpersonal differences observed. In the human plasma proteome, highly abundant proteins, such as serum albumin and transferrin, do not show high intra- and interpersonal differences. However, in the case of human tear fluid, highly abundant proteins, such as lactoferrin, lysozyme, and lipocalin, which represent approximately 80% of the whole protein amount, demonstrate high variances. The calculated interpersonal variances can be compared with that observed in the saliva, with both the TPC and the amount of the major saliva proteins (amylase and immunoglobulins) displaying high interpersonal variances [35]. Intra- and interpersonal variations in the absolute concentrations of 90 saliva proteins, as determined by targeted LC–MS, are on a similar level (45% and 69%, respectively [31]) to that observed in our study of tear fluid.

For the possible identification of the relations of the quantified proteins to global tear characteristics, the correlation between the quantified proteins with TPC and tear volume was examined. In general, no strong correlation was found between measured individual protein amounts and either total protein content or tear volume.

#### 2.3.3. Methods to Decrease Variance: Normalization and Selection of Representative Samples

Different normalization approaches can be generally used to balance for intensity variations related to technical issues in mass spectrometry. We have applied the NormalyzerDE 1.5.4 [36] software to evaluate different normalization methods. Possible tear household proteins were also selected for normalization. Immunoglobulin A was proposed as a continuously secreted potential household protein in the DEWS II paper [37] for normalization of tear volume. The average of the intensities of the two most abundant proteins with low variance (LTF and LCN1) was also evaluated as a potential normalization factor. Based on our conclusions on protein cluster analysis discussed in the next section, cluster normalization using four clusters was applied in addition to the above-mentioned methods. Cluster normalization was found to be the most effective based on measures of performance calculated (Supplementary Table S4 and Figure S5). Even with such normalization, intrapersonal mean variance and sample-to-sample correlation were not satisfying if all samples were included (Table S4). It must be noted that normalization based on selected proteins (IgA, LTF/LCN1) actually increased the median variance of proteome composition, caused by an artificial increase in the variance of other proteins, changing independently from these highly abundant proteins.

**Figure 2:**
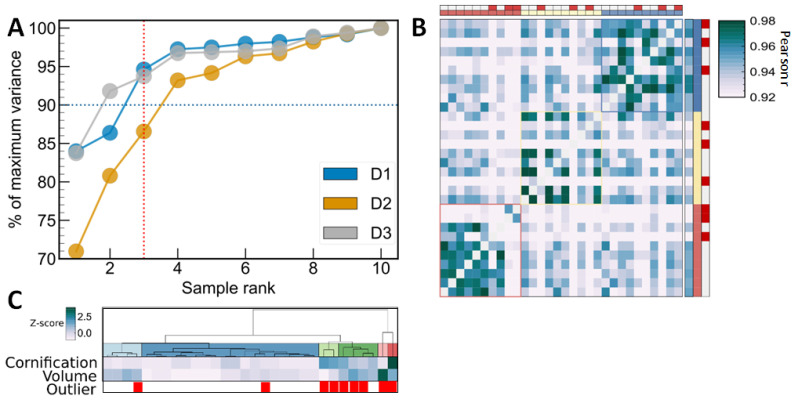
Identification of representative personal sample sets based on leave-one-out variance elbow method (**A**) and heatmap of sample-to-sample Pearson correlation (**B**). Average intrapersonal Pearson correlation coefficients for each sample are shown on the right. Outlier samples are marked with red color on edges. Heatmap of summed intensity of cornification-related proteins (e.g., keratins) and tear volume are shown as possible classifiers of outlier/representative samples (**C**). Summed intensities of proteins with ‘Cornification’ GO Biological process assignment and tear volume were z-score normalized before hierarchical clustering of samples.

Because of the complex nature of tear fluid (different liquid layers, possible mixture of reflex and basal tear, etc.), variance may be introduced during sample collection. In some cases, this may cause collection of outlier samples not representative of personal proteome profile. We have evaluated traditional methods for identification of representative samples based on intrapersonal correlation (Figure 2) and principal component analysis (Supplementary Figure S5). Using these methods, it may be difficult to determine thresholds for correlation coefficients or confidence intervals, thus the number of samples to be excluded from analysis. The effect of exclusion of these samples on intragroup variance is not predictable by these approaches, and therefore we have introduced an elbow method based on a leave-one-out coefficient of variances (L1OCV). Figure 2A represents the method: the median intrapersonal protein intensity variance calculated after leaving one sample out (L1OCV) relative to the maximum L1OCV as a function of sample rank on L1OCV. Samples ranked first this way are those having the largest effect on intragroup variance and, based on the vertical axis, the actual improvement of CV. The optimal number of outliers, which have significant effect on variance, can be visually determined from the break-down point (elbow) of the curve (three samples in this case) or choosing a relative L1OCV limit (e.g., 90%). L1OCV values were calculated using the MSDAP R package [38]. Using our controlled sample collection protocol, performed by the same person, 10–30% of samples were found not to be representative of the personal proteome, and thus can be classified as outliers.

Even in the case of high-abundance proteins, intensities and protein ranks are altered in a complex way compared to representative samples, which explains the low performance of most global normalization approaches. It must be noted that seven out of the nine outlier samples were collected with high tear flow (above personal median measured volume, Figure 2C), so formation of reflex tear cannot be excluded in these cases. Tear volume and total intensity of cornification-related proteins (e.g., keratin) were significantly higher in outlier samples, so these values may serve as preliminary quality control, if normal levels can be determined in the experimental setup.

#### 2.3.4. Contribution of Proteoforms to Observed Variance

**Figure 3:**
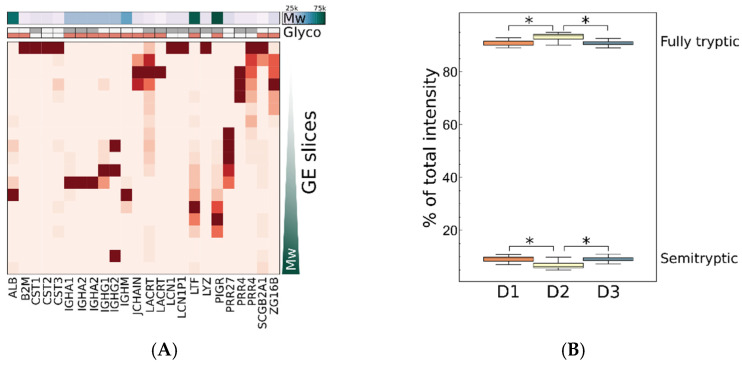
(**A**) Representation of distribution of proteins detected in more than two out of 19 GE bands. Detection is shown by squares colored according to spectrum count in the specific gel slice. Theoretical molar masses calculated for mature forms of proteins are shown on the top (Mw). Glycosylation from Uniprot annotation or reference [39] (red) and MS/MS identification from this work (grey) are shown on the top (Glyco). Repeated gene names represent identified protein variants. (**B**) Relative intensity of fully tryptic and semitryptic peptides measured in all quantitative samples of each individual. Significantly different (*t*-test, *p* < 0.05) interpersonal levels are marked with an asterisk.

In the general expressional proteomics LC–MS identification protocols, databases of canonical protein sequences are used, and a low number of frequently occurring post-translational or chemical modifications are considered assuming strict enzyme specificity. Asthe majority of the identified proteins are known to be glycosylated and/or phosphorylated according to the Uniprot database, we investigated whether variations in the level of PTM contribute to the observed variance in quantification based on unmodified peptides. On the other hand, we have identified 35 different proteases in the tear samples; therefore, proteolysis occurring with specificity other than that of trypsin may also contribute to variations in the amount of detected tryptic peptides. For the building of spectral libraries, we have performed SDS PAGE separation of proteins, so while we could not just analyze the individual quantitative samples for modified or semitryptic peptides, we could identify proteins that were found in more than one non-consecutive GE band. Among other reasons, extensive non-uniform glycosylation, the presence of different endogenous proteoforms, or proteolysis may cause such electrophoretic behavior. Figure 3 shows GE band distribution of those proteins which occurred in more than two bands.

**Figure 4:**
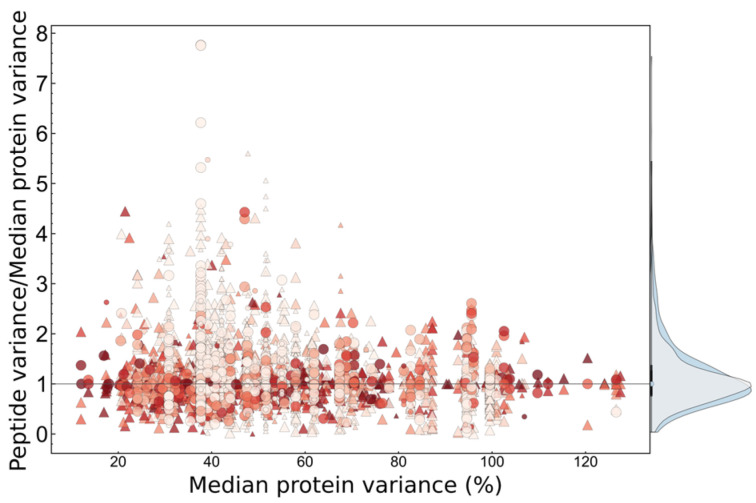
Variance of peptides relative to median peptide variance of assigned protein. Modified peptides (phosphorylated or glycosylated) are represented with triangles. Semitryptic peptides are shown with small markers. Points are colored according to relative intensity (e.g., dark red points represent the most intensive peptides of the specific protein). Relative CV distributions of semitryptic (grey) and fully tryptic (blue) peptides are shown on the right.

Proteins detected in multiple GE slices were mostly glycosylated and were identified by both tryptic and semitryptic peptides, thus various explanations of electrophoretic migration may exist. Peptide fragments of most of these proteins were identified in previous peptidomic analysis of tears [40], which confirms their possible proteolysis. We have found lacritin- and proline-rich protein 4 to be spread over the widest gel area, which is in accordance with the high number of peptide fragments of those proteins in human tear peptidome [40], and the known proteoform diversity of lacritin [41]. The identification of specific proteoforms and PTM sites was outside the focus of this work; we simply evaluated the effect on quantification, which could be most effectively studied at peptide level. We have observed a quite constant total semitryptic/tryptic ratio for each person, with around 90% of total intensity originating from tryptic peptides, but significant interpersonal difference was observed in that number (Figure 3B). This may be a consequence of different levels of proteases, but also tear flow rate may have an effect on it by turnover rate of tear film. Total intrapersonal variance, however, depends on protein entity; therefore, in order to analyze individual peptides and put them on the same scale, variances were normalized to median variance of all peptides of the actual protein. Peptides of a protein with extraordinary variance can be identified this way. Figure 4 represents this relative variance as a function of median peptide variance of proteins, and relative variance distribution of fully tryptic and semitryptic peptides. We observed no significant difference in intrapersonal variance distribution of semitryptic/tryptic and modified/unmodified peptides. Therefore, we can conclude that, globally, the measured intrapersonal variance is not significantly affected by proteoforms, but for specific proteins there may be such effects. On the other hand, significant interpersonal differences could be found in the intensity of some modified and semitryptic individual peptides and total level of semitryptic peptides (Figure 3), and therefore in interpersonal comparisons these effects should not be neglected. PTM-related variations [28] and peptide-dependent reproducibility (Figure 4) should be considered, especially in peptide selection for targeted LC–MS analysis.

#### 2.3.5. Statistical Determination of Sources of Biological Variance

In order to find protein groups with similar function or origin, protein–protein correlation and hierarchical clustering analyses were performed.

**Figure 5:**
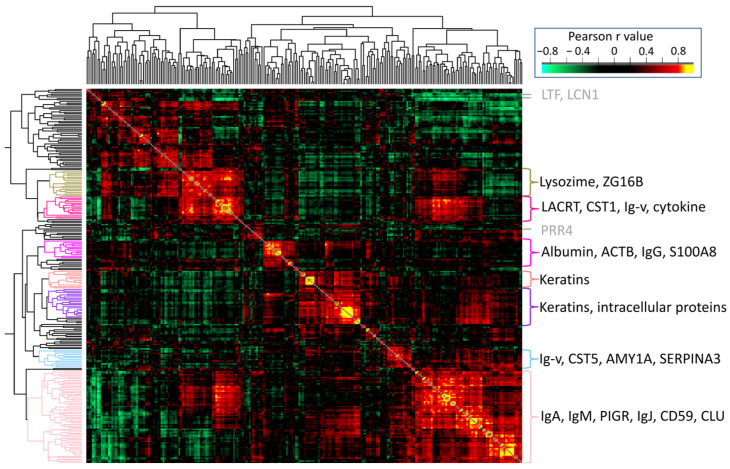
Protein–protein correlation heatmap after hierarchical clustering based on Pearson correlation coefficients. Clusters including members with r > 0.9 are colored, and characteristic/highly abundant proteins/groups are shown on the right. Highly abundant proteins not assigned to any of the highlighted clusters are also marked with gene names in grey.

The heatmap representing protein clusters with similar correlations can be seen in Figure 5, while data can be found in the correlation matrix in Supplementary Table S5. We have identified several major protein clusters, in which the proteins showed strong (Pearson r > 0.8) or intermediate correlation (r > 0.5). The largest cluster is made of different variable Ig chains, IgA, IgM, and some enzymes serving as defense. Keratins and intracellular (e.g., nuclear) proteins form the second largest cluster. Serum-derived proteins (albumin, S100A8, and IgG) form another strongly correlated cluster, while the cluster including major tear proteins lysozime, lacritin, and cytokins also shows positive correlation to some proteins of the major Ig cluster. Contrary to this, some highly abundant proteins, such as lactroferrin, lipocalin, and proline-rich protein 4, do not show strong correlation with any larger cluster of proteins. The observed positive correlations may be due to common function, biochemical control, or origin of the proteins. Immunoglobulin chains may be present as covalently bound complexes in the samples [42], therefore their abundance may be strongly correlated; however, there are several sources of Ig content in tear fluid, which explains diverse correlations to other tear proteins. Keratins are considered normal components of tears because of the contact between this fluid and the patient’s skin, and they enter tear fluid together with cellular debris; thus, the abundance of different keratins and intracellular proteins may be correlated, as was found. Correlation and clustering of lower-abundance proteins with less knowledge of functions and origin may aid identification of their source role in tear fluid.

**Figure 6:**
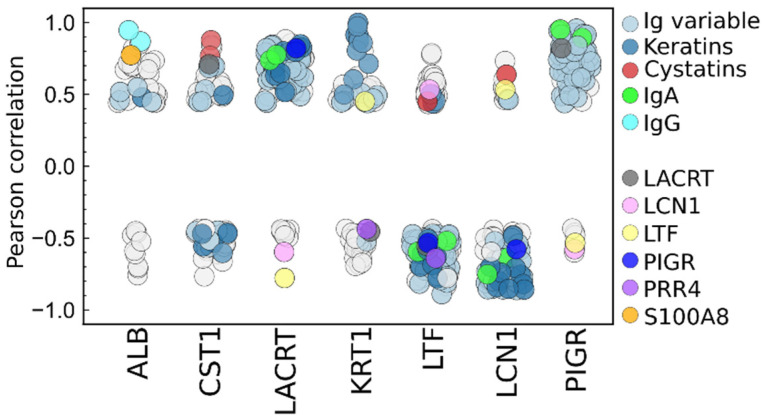
Protein–protein correlation (Pearson correlation coefficients, r) of selected key highly abundant tear proteins. Only significant (*p* < 0.05) correlations are shown for each protein. Specific protein groups and individual proteins are colored.

To visualize positive or negative correlations between major proteins of the observed clusters, significant (*p* < 0.05) Pearson correlation coefficients of those are shown in Figure 6. Levels of serum albumin, IgGs, and S100A8 show strong correlation to each other, and vary independently from major tear proteins, which confirms their serum origin [43]. The defensive enzymes, cystatins, are strongly correlated with each other, while their abundance showed significant interpersonal differences, as was shown in the previous section. The highly abundant tear protein lacritin is strongly correlated with major Igs (IgA, PIGR, and most Ig-v chains) but shows a relatively strong negative correlation to other major tear transport proteins lactoferrin and lipocalin. Lacritin is known to modulate secretion by lacrimal acinar cells, down-regulated in dry eye syndrome [44], which are in agreement with its positive correlation with the continuously secreted IgA. It must be noted that the strong negative correlations may be a consequence of the normalization of total protein content (constant protein amount digested), and therefore, if the relative abundance of a protein is increased, there should be a decrease in other proteins. This effect is mainly visible in correlation coefficients of highly abundant proteins, and if variances are low enough, the negative correlations may be considered as signs of independent control of expression/secretion and, in the case of tears, as an indication of origin from different members of the lacrimal apparatus.

#### 2.3.6. Interpersonal Comparisons Based on Replicate Personal Samples

**Figure 7:**
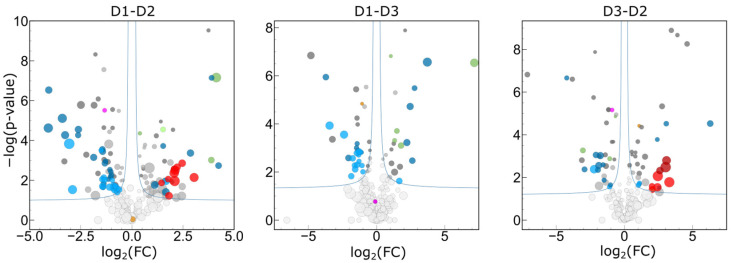
Volcano plots of the differential analysis of normalized protein intensities for all individual comparisons. −log_10_(*p*) values of *t*-test are shown as a function of log_2_(Fold change) for each protein. Significance limits determined based on sample randomizations (FDR < 0.05) are shown with blue lines. Size of dots represents intrapersonal variance. Significantly different keratins (red), Igs (blue), and cystatins (green) are colored, lighter colors representing those proteins which were significantly different, only if analysis was limited to representative samples. Two proteins with personal profile are also colored: the putative reflex tear marker proline-rich protein 4 (PRR4), is shown with violet, while Ceruloplasmin with orange dots.

Considering the large intrapersonal variances described in the previous sections, it had to be confirmed whether statistically significant interpersonal differences can be detected in the tear proteome of ophthalmologically healthy individuals. We have performed Student’s *t*-test in all possible two-sample comparisons, applying a strict randomization-based significance limit (FDR < 0.05), both using all samples (*n* = 10/person) and limited to representative samples (*n* = 7/person). Analysis limited to representative samples resulted in, on average, 30% higher significantly different quantifications (Figure 7), confirming the importance of quality control. The most important protein classes with significantly different interpersonal abundances are different Ig chains, keratins, and cystatins. Sixteen proteins were found to be significantly different in all three comparisons, including five Ig chains and three cystatins. All five cystatins and 29 Ig chains were among the 90 proteins significantly different in at least two comparisons. Different Ig chains and Cystatin SN were recently found to be highly down-regulated in tear samples of patients with meibomian gland dysfunction [45], therefore the observed interpersonal variance of these proteins may be a consequence of individual activities of different glands secreting the tear proteome. We have measured increased average tear volumes from one eye of both D1 and D3, thus the increased keratin levels in tears of these subjects may be related to the higher tear flow. The level of proline-rich protein 4 (PRR4), which was found to be a reflex tear marker [15], was also found to be different in the samples of these subjects, compared to those of D2. Abundance of ceruloplasmin (CP) was found to be significantly higher in samples of D3 (female) relative to both D1 and D2 samples (males), which is the only observed possible gender-related difference; however, other factors may also be responsible for this (e.g., tear levels of CP were found to increase with age [46]). We have not observed sex-related differences in the global proteome profiles (e.g., PCA analysis on Supplementary Figure S6) and levels of individual intrapersonal/intra-eye variances. It must be noted however, that the identification of reasons for individual protein levels was outside the focus of this study, and the limited number of participants and the lack of complete medical records does not allow the drawing of any conclusions on the origins of differences. Our pilot study, however, may serve as a proof of concept for the comparison of individual tear proteomes using repeated sampling.

While eyes are paired organs, they can react differently to stimulus, so the personal inter-eye differences were also investigated. Paired *t*-tests at the eye level (five sample pairs for each person) did not identify any significant differences between two eyes of any person using the same methods applied in interpersonal comparisons. This is, however, not the consequence of lower sample size, as significant differences could be identified between samples from single eyes of different subjects.

In our samples, 72 different immunoglobulin chains were identified, 53 of which could be quantified in all samples. Our interpersonal comparisons also identified several significantly different Ig chains, and recent reports on possible application of specific immunoglobulins [47] as biomarkers in different diseases emphasize the importance of studying the composition of this protein class. Total level of Igs varied sample-to-sample, therefore their intensities were normalized to the sum of intensities of all Igs in this analysis, to obtain better correlations.

**Figure 8:**
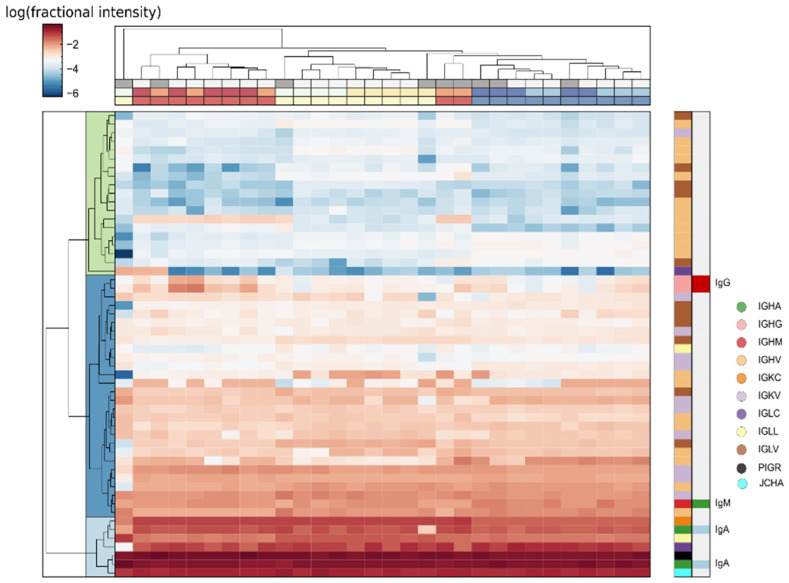
Hierachical clustering of samples based on relative Immunoglobulin intensity normalized to total Ig intensity. Classification of Ig chains (A/G/M, heavy/light, constant/variable) is shown on the right. Personal and eye assignment of samples are denoted by colors (red/blue/yellow) on the top. Outlier samples are marked grey at the top.

Ig composition was found to be relatively stable, and the top five Ig chains were the same in all the samples, except for some samples which were previously assigned as outliers (Figure 8). All the personal samples were clustered based on Ig composition, except for some outlier samples, reflecting the existence of a stable personal Ig profile. Differential expression analysis also identified numerous Ig chains as significant interpersonal differences, as described previously. Based on protein correlations, most Igs show strong correlation with major tear proteins TRFL and LCN1, except for IgG-heavy chains (IGHG1 and IGHG2), which are negatively correlated with those, and strongly correlated with serum albumin. This is in accordance with the known origin of these Igs (serum for IgG and lacrimal glands for IgA) [43].

Based on the findings for the Ig sub-class and the relative effectiveness of cluster normalization, it is possible that a similar approach, involving independent normalization and analysis of specific, biologically, or statistically selected protein groups or clusters may lead to a more effective differential expression analysis.

## 3. Materials and Methods

### 3.1. Participants

Healthy individuals with no concomitant systemic or ocular diseases were recruited for this study. The study participants did not use systemic or topical ocular medications. The general medical and ocular histories of the participants were unremarkable.

### 3.2. Ophthalmological Examination

All participants underwent a detailed ophthalmological examination that included visual acuity testing, intraocular pressure measurement, evaluation of tear volume using Schirmer strips (without anesthesia), ocular surface staining with 2.0% sodium-fluorescent dye, measurement of tear film breakup time by a slit lamp (under a cobalt blue filter), and indirect ophthalmoscopy. The fluorescein breakup time was evaluated after the instillation of 0.5% sodium-fluorescein. The subjects were subsequently asked to blink several times. The time in seconds between the last complete blink and the appearance of the first corneal black spot was measured. A standard Schirmer test strip was then placed in the lateral canthus for 5 min with the eyes closed. The length of strip wetting was measured using a millimeter scale.

### 3.3. Sample Collection

Sample collection complied with the guidelines of the Helsinki Declaration, and ethical approval was obtained from the University of Szeged Ethical Committee (108/2019-SZTE). All participants provided written consent to participate in the study. Samples were collected from three non-smoking healthy volunteers (D1, D2, and D3) using the glass capillary method (Harvard Apparatus Ltd., Cambridge, MA, USA). Janssen and his coworkers examined the influence of the sampling point, sample collection method, and difference in the protein content of the left and right eyes of the participants and found significant differences in the tear protein composition [48]. According to their results, the tear samples collected from the outer eye corner contained the lowest amount of serum proteins. Therefore, samples were collected from that part of the eye in the current study on five consecutive days without touching the eye. The capillary method was our preferred choice to characterize the tear fluid, to avoid surface protein contamination caused by direct contact and protein adsorption on the Schirmer strips. According to the literature, many factors can affect tear secretion. Thus, during the sample collection, we attempted to set the humidity, brightness, stimuli, sampling point, and participant position in a consistent manner. However, the sample collection itself may provide a stimulus; therefore, it is considered difficult to reproducibly collect pure basal tear samples. To address this issue, a simple, mild, reproducible stimulus of wiping the skin underneath the targeted eye with ethanol was applied in this study.

### 3.4. Sample Processing

After sample collection, tear samples were transferred into Eppendorf tubes and centrifuged at 12,000 rpm at 4 °C for 5 min. The samples were then aliquoted, frozen, and stored at −80 °C until analysis. Whole tear volumes were measured and the total protein concentration (TPC) of each sample was determined using the Qubit Protein Assay Kit (Thermo Fisher Scientific, Waltham, MA, USA) following the manufacturer’s protocol. In total, 30 individual tear samples were collected, processed, and analyzed. For a summary of the arrangement of the collected samples, their analysis, and statistical grouping, see Supplementary Figure S1.

In order to build a more comprehensive peptide library, SDS-PAGE pre-fractionation was performed on a pooled sample. The gel was divided into 19 pieces, and the proteins were in-gel digested. Individual tear samples, containing 1 μg protein, were in-solution digested by using a modified version of Turiak’s mini protocol [49].

To extend the protein library built from the gel-separated pooled sample, three tear samples were created by mixing equal amounts of all the samples originating from the same person. These pooled samples of each individual were digested with an ‘in solution’ digestion method.

For the determination of the technical variance, digestion of three replicates of a randomly chosen tear sample was performed, and each digested sample was analyzed by LC–MS in triplicate.

### 3.5. LC–MS Analysis

The separation of the digested samples was carried out on a nanoAcquity UPLC, (Waters, Milford, MA, USA) using Waters ACQUITY UPLC M-Class Peptide C18 (130 Å, 1.78 μm, 75 μm × 250 mm) column with 90 min gradient. Eluents were water (A) and acetonitrile (B) containing 0.1 *v*/*v*% formic acid, and the separation of the peptide mixture was performed at 45 °C with a 0.35 μL/min flow rate.

In order to reduce the coelution of peptides and maximize the number of identifications, an optimized LC gradient was used. LC–MS data obtained from our standard 90 min linear gradient (3–40% B) served as an input to GOAT Version 1.0.1 gradient optimization software [50], and the optimized gradient elution was applied in further analysis. The LC was coupled to a high-resolution Q Exactive Plus quadrupole-orbitrap hybrid mass spectrometer (Thermo Scientific, Waltham, MA, USA). For measurements to build the protein library, the mass spectrometer was operated in DDA (Data Dependent Acquisition) mode.

The “fast” method from Kelstrup [51] was used for DDA acquisition. The mass spectrometer was operated in the data dependent mode to automatically switch between full scan MS and MS/MS acquisition. The full scan was performed between 300 and 1750 *m*/*z* with 70,000 resolution at 200 *m*/*z*. The MS/MS scans were carried out with 17,500 resolution after accumulation of ions to a 1 × 10^6^ target value based on predictive AGC from the previous full scan. The 12 most intense multiply charged ions were fragmented in the octopole collision cell by normalized HCD collision energy (25%). The spray voltage was set to 1.8 kV, and the capillary was heated to 275 °C. The MS/MS ion selection threshold was set to 1 × 10^5^ counts.

The quantitative measurements of digested individual samples were performed in DIA (Data Independent Acquisition) mode. The survey scan for the DIA method operated with 35,000 resolution. The full scan was performed between 380 and 1220 *m*/*z*. The AGC target was set to 1 × 10^6^ or 120 ms maximum injection time. In the 400–1200 *m*/*z* region, 32 windows of 25 *m*/*z* width were acquired at 17,500 resolution (AGC target: 3 × 10^6^ or 100 ms injection time, normalized collision energy: 30 for charge 2).

### 3.6. Data Analysis

To study the inter- and intrapersonal differences, a comprehensive spectral library was built from DDA runs (pooled samples and gel bands).

The DDA spectra were processed with FragPipe (version 19.0) analysis software [52]. The minimal peptide length was set to 6. Search criteria included carbamidomethylation of cysteine as a fixed modification, with oxidation of methionine and acetyl (protein N-terminus) as variable modifications. The mass tolerance for the precursor was 20 ppm and for the fragment ions was 20 ppm. The DDA files were searched against human proteome from the UniProt database (2022.12, 71,785 entries). Trypsin was defined as enzyme with a maximum of two missed cleavages allowed using 1% FDR limits both on peptide and protein level. The minimum peptide length was 7. The quantitative analysis using the FragPipe search output library was performed in DIA-NN (version 1.8.1) [53]. The statistical evaluations and data normalizations were carried out using Perseus (version 1.6.14) and Instantclue 0.11.2 [54] software on text reports from DIA-NN (version 1.8.1) software. NormalyzerDE 1.5.4 [36] was used to evaluate different normalization approaches. MS-DAP 1.02 was used for quality control and calculation of leave-one-out coefficients of variation [38].

To quantify the glycoforms and the phosphorylated and nontryptic peptides, and to study their possible variance contribution, additional libraries were built in FragPipe. Closed search was applied either specifying phosphorylation on serine, threonine, or tyrosine assuming fully tryptic peptides, or semitryptic digestion specificity on the database of previously identified proteins and contaminants (572 entries). Glycoforms were identified using mass offset workflow, using common glycosylation mass adducts. Glycosylation identifications were manually validated by the presence of glycosylation reporter ions. In all cases, 1% FDR limits were applied. Example MS/MS spectra are shown in Supplementary Figure S7.

Identified proteins were annotated by Gene Ontology terms and keywords from the UniProt database (https://www.uniprot.org (accessed on 12 April 2022)).

For the calculation of the different levels of variance, samples were grouped as follows: for intra-eye variance, the protein profiles through five days of the two eyes were treated as two different groups for each person. For the determination of intrapersonal variance, all samples of the same person (including the samples of both eyes) were treated as one group. For the estimation of interpersonal variances based on the same sample size, samples were divided into three groups randomly. Within each random group, a similar number of samples from each individual was included. To evaluate the overall variance, all samples (including all the samples of all donors) were treated as one group. For a summary of statistical grouping of samples, see Supplementary Figure S1.

### 3.7. Materials/Reagents

Calibrated glass microcapillary tubes (20 mL) were manufactured by Drummond Scientific Company (Broomall, PA, USA).

Reagents, such as ammonium bicarbonate (ABC), dithiothreitol (DTT), iodoacetamide (IAA), and sodium dodecyl sulfate (SDS) were purchased from Sigma-Aldrich (Darmstadt, Germany), acetone from Merck (Darmstadt, Germany), trypsin and formic acid (FA) from Thermo Scientific (Rockford, IL, USA). Water, acetonitrile, and acetic acid were delivered by VWR (Debrecen, Hungary).

## 4. Conclusions

In this study, a quantitatively reproducible digestion protocol was optimized for the limited protein content of individual tear samples (0.1–0.3 μL), which enabled LC–MS/MS measurements without pooling the tear samples. In order to maximize the number of identified and quantifiable proteins, the LC gradient and the MS acquisition parameters were also optimized.

Considerable intra- and interpersonal variances were found in the tear volumes and the total protein content of the samples. Significant differences were detected in the protein compositions of samples from the same person depending on the choice of eye (left/right) and day of sample collection. We observed intra- and interpersonal variances using glass capillary sample collection at similar levels to those of Schirmer’s strip samples [28], although sampling adds an additional level of variability, which is hard to differentiate from those of biological/medical origin. Based on these observations, some suggestions can be made to increase the statistical significance of biomarker discovery experiments with variable body fluids, such as tear fluid or saliva. The number of persons included in the analysis and the minimum acceptable fold change should be carefully chosen. Our results demonstrate that interpersonal comparative analysis of samples from single eyes may lead to different conclusions than samples from both eyes. The collection of several samples (both eyes, different days) from each person (analyzed either separately or in a pooled fashion) is recommended. If pooling is chosen, based on personal and eye-specific normal tear flow rate, outlier samples can be excluded before any LC–MS analysis. In all cases, experimental designs should take into consideration a high expected level of intra- and interpersonal variance to obtain statistically meaningful results. It must be emphasized that consistency in sample collection is essential for tear proteome profiling.

Intrapersonal and sampling variations in human tear samples may raise problems in biomarker identification and diagnosis. We have introduced a method for the identification of representative samples, which provides direct estimation of the number of outlier samples and improvement of the coefficient of variance. Variance can only be decreased to an acceptable level by sample filtering, as most normalization methods fail to compensate for the compositional variance of tear samples. Normalization performed separately within groups/clusters of proteins may be a viable route to decrease observed variance, but global normalization based on selected proteins should be avoided. Despite the high intrapersonal variance, statistically significant differences can be identified in the tear protein profiles of even ophthalmologically healthy individuals, especially in proteins responsible for defense. The significance of such analysis can be increased if representative samples are selected, widening the possible applications of tear samples in personal and predictive medicine.

At least some part of the observed variance is normal, however, considering the dynamic and multiplex nature of tears, so studying tear fluid as a dynamic system may help us to better understand some biochemical aspects of this body fluid, and offer better control of sample variance. Differences in the protein composition of tear samples from the two eyes of a subject should be considered in any study; in our view, no single eye or single time sample can be considered as personally representative. Future studies to classify proteins based on variations due to different stimuli and/or different sample collections may help to identify the source and function of individual proteins and protein classes in tears. Identification of variance introduced by sampling of tear fluid is a great challenge, but detailed analysis of this aspect may provide additional biochemical insights too. A personal immunoglobulin profile was recognized after normalization to total IG content, and different sources of IgG (serum) and IgA/IgM (lacrimal gland) were confirmed, based on correlation analysis. Moreover, the strong correlation of IgA1, JCHAIN, and PIGR confirmed that the majority of IgA is present in polymeric form [42,55], while variations in IgA1/IgA2 ratio were also observed. We conclude that the addition of body fluid-specific protein annotations besides the general Gene Ontology to databases could help in the interpretation of body fluid proteomics results.

## Figures and Tables

**Table 1:** Volunteers’ detailed demographic data and results of ocular surface examinations (BUT: break up time; OD: oculus dexter/right eye/; OS: oculus sinister/left eye/).

	Age/Gender	Schirmer I Test OD/OS	BUT OD/OS	Corneal Fluorescein Punctate Staining
D1	26/male	25/25	25/25	no
D2	27/male	20/20	20/25	no
D3	33/female	20/20	25/23	no

## Data Availability

The spectral library and quantitative results from Encyclopedia analysis are available through Zenodo at: https://doi.org/10.5281/zenodo.10498136 (MS data) and https://doi.org/10.5281/zenodo.10498289 (Results) (accessed on 12 January 2024).

## Supplementary Materials

The supporting information can be downloaded at: https://www.mdpi.com/article/10.3390/ijms25031559/s1.

## Abbreviations

AGC	Automatic gain control
ALBU	Albumin
ANOVA	analysis of variance
BUT	break up time
CIS	clinically isolated syndrome
CV	coefficient of variation
D	Donor
DDA	data dependent acquisition
DIA	data independent acquisition
DTT	dithothreitol
EyeOME	The Human Eye Proteome Project
FDR	false discovery rate
HCD	Higher-energy collisional dissociation
IAA	iodoacetamide
Ig	immunoglobulin
LC/MS	Liquid chromatography–mass spectrometry
*m*/*z*	mass/charge
M	mol/L
MS/MS	tandem mass spectrometry
MS	mass spectrometry
NH_4_HCO_3_	ammonium-hydorgencarbonate
OD	right eye (oculus dexter)
OS	left eye (oculus sinister)
Pers	personal
PIGR	Polymeric immunoglobulin receptor
SDS-PAGE	sodium dodecyl sulphate–polyacrylamide gel electrophoresis
Tot	total
TPC	total protein concentration
TRFL	lactotransferrin
UPLC	Ultra Performance Liquid Chromatography
*v*/*v*%	volume/volume %
Vol	volume
