# Supplementary material for: Sources of Variance in Human Tear Proteomic Samples: Statistical Evaluation, Quality Control, Normalization, and Biological Insight
Source: Int J Mol Sci. 2024 Jan 26;25(3):1559. doi: 10.3390/ijms25031559 (PMC10855525; doi:10.3390/ijms25031559)

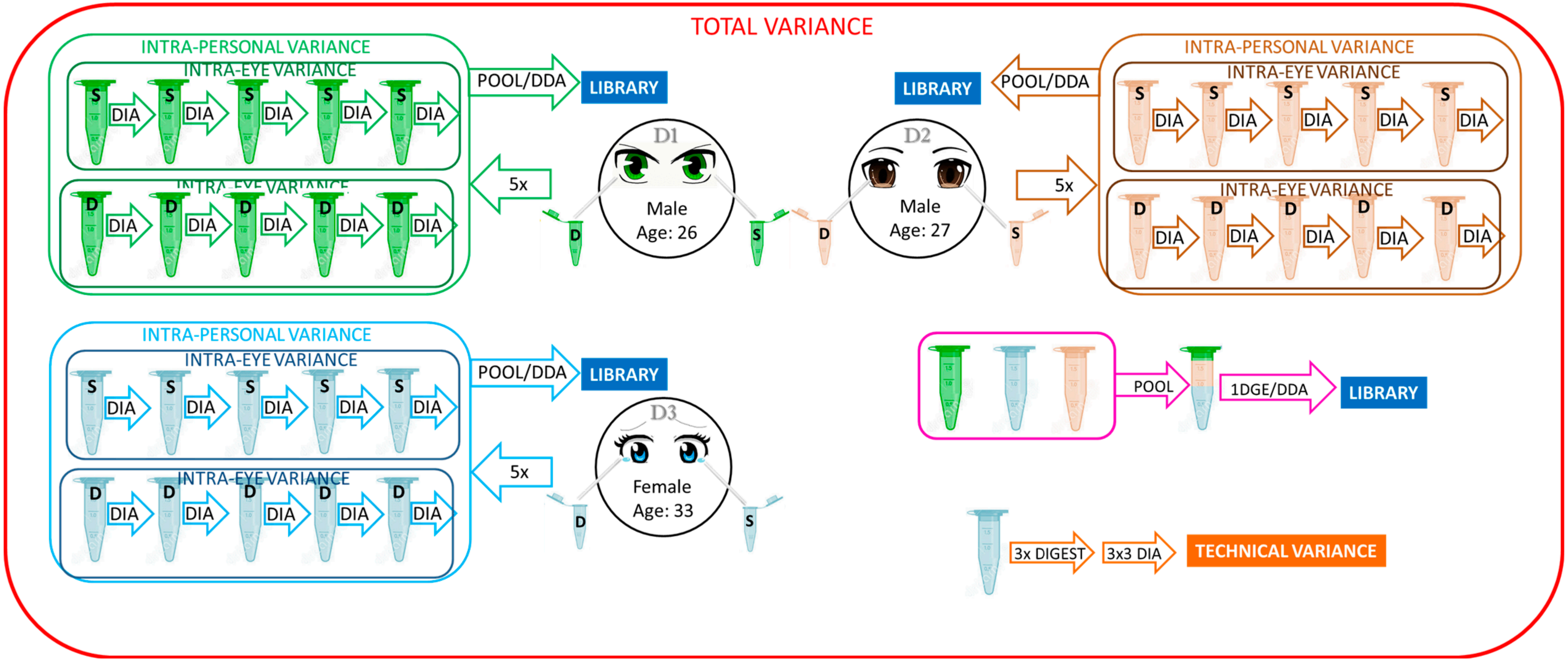

Figure S1. Graphical represantation of samples, measurements and statistical sample grouping

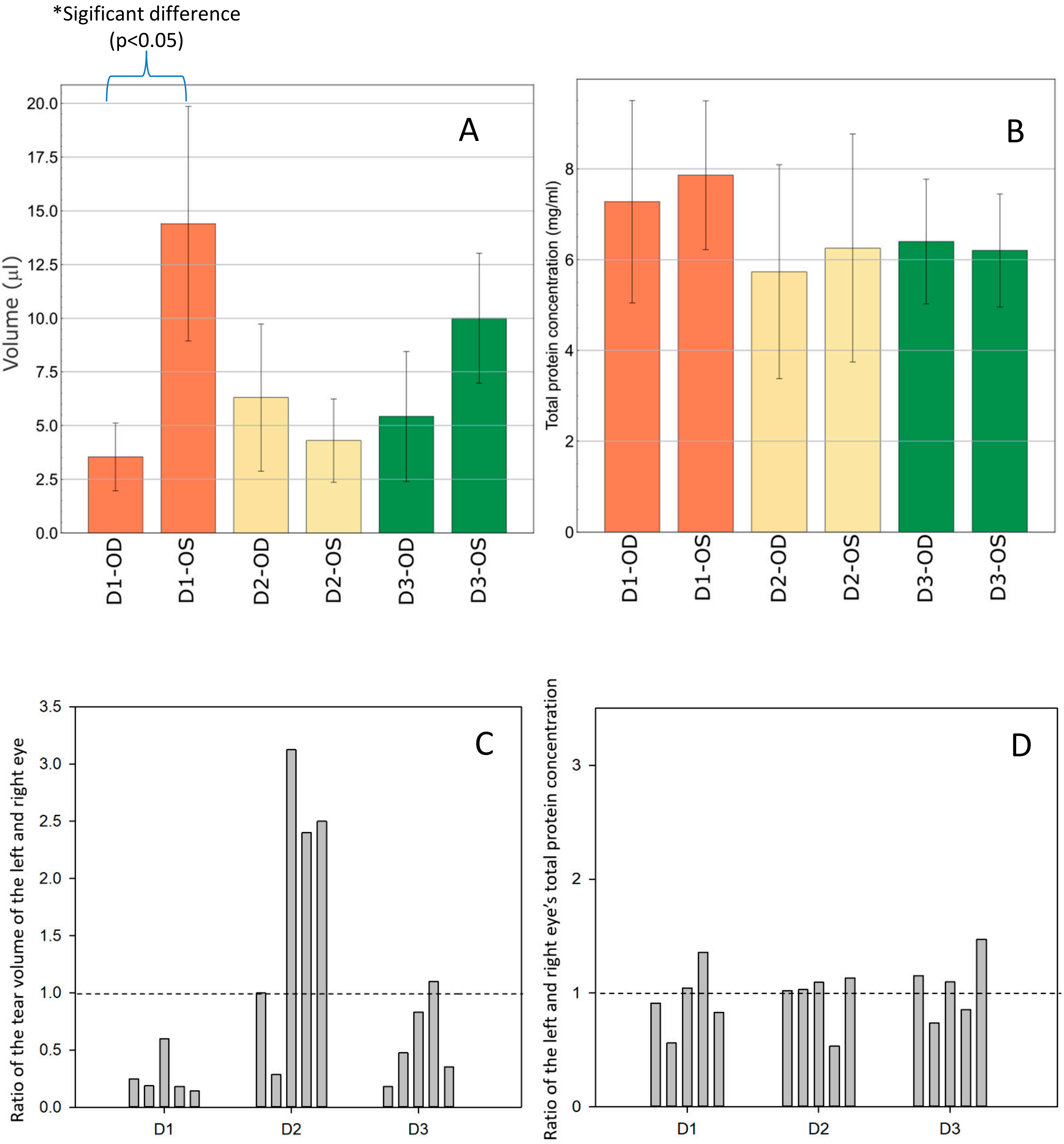

Figure S2: Barplot representation of the intraeye personal variances in the tear volumes (μL) collected in 2 min (A) and the total protein concentrations (B) (μg/μL) of the right eye (OD: oculus dexter) and left eye (OS: oculus sinister) for each participant (D1, D2, and D3).

Daily left eye/right eye ratios of the tear volume (C) and TPC (D).

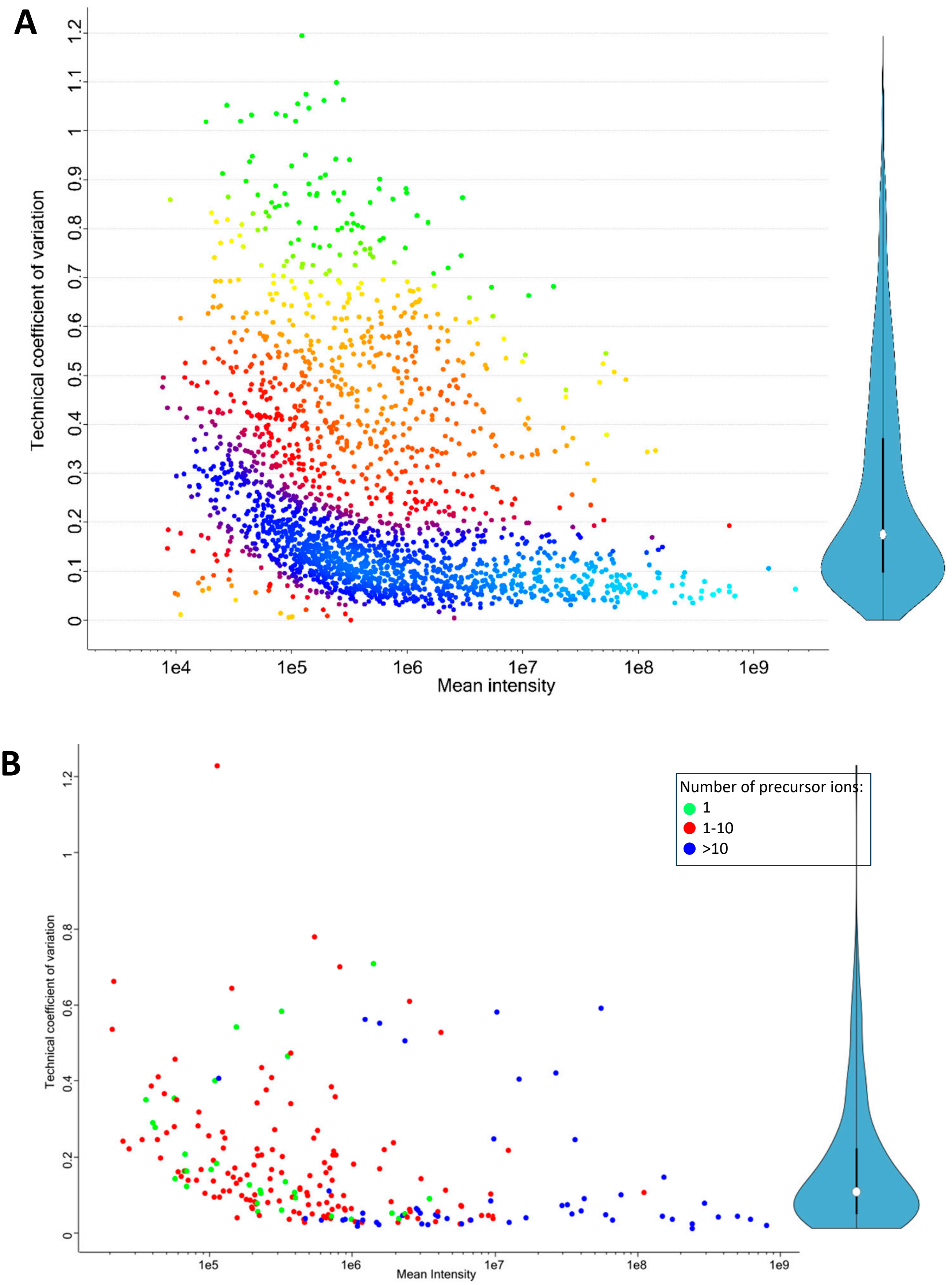

Figure S3.

Technical variance distribution of measured peptide (A) ion and calculated protein (B) intensities in triplicate LC-MS analysis of three repeated digests of a selected sample. Dots are coloured based on distribution density (A) or number of precursor ions (B). Distribution is shown on the right as a violinplot, median is shown with white dot.

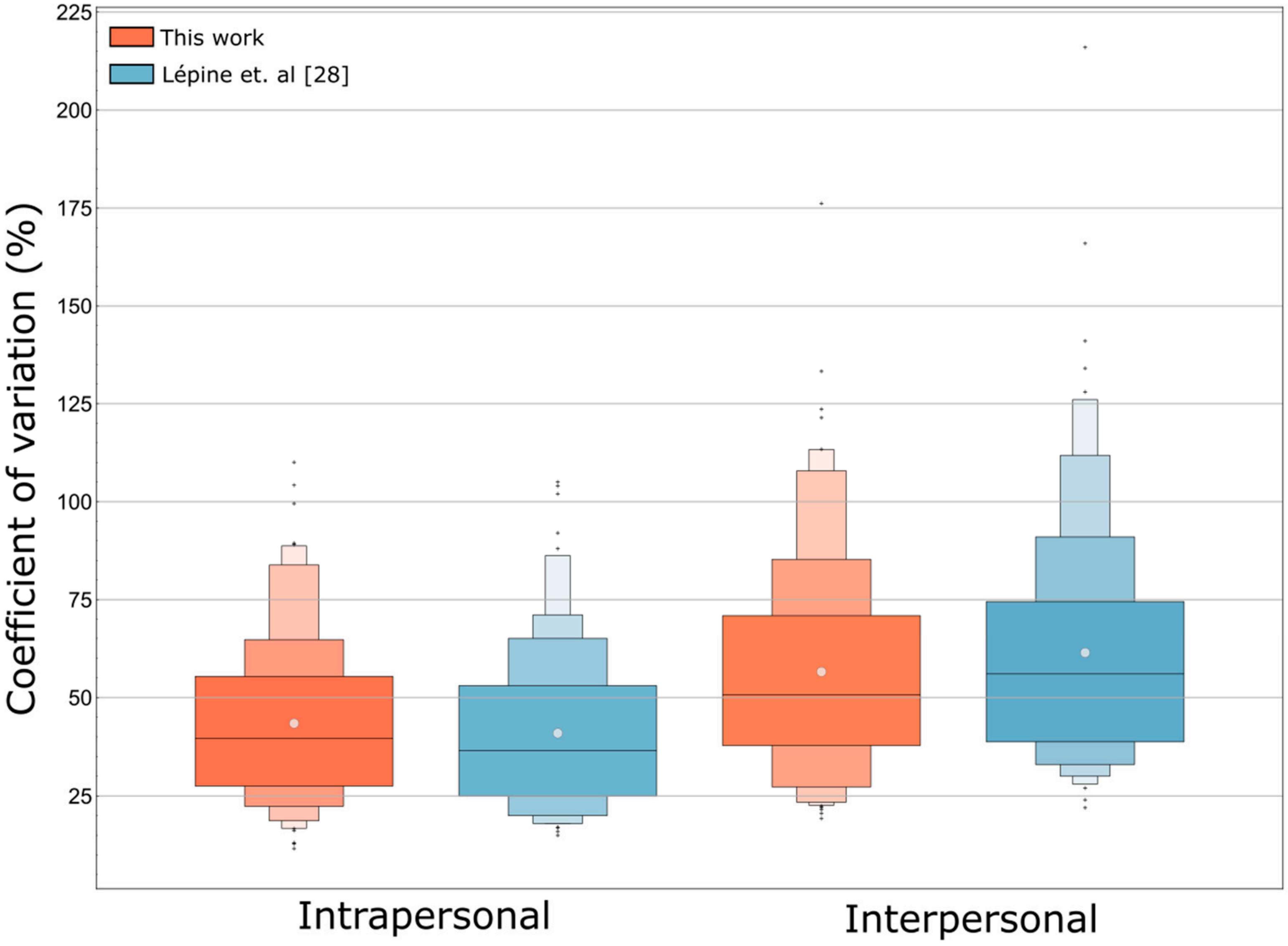

Figure S4. Intrapersonal and interpersonal coefficient of variation (%) distribution of common proteins identified in this study from capillary samples and in paper of Lépine et. al. [28] from Schirmer's strip samples. Intensities were normalized to total protein intensity.

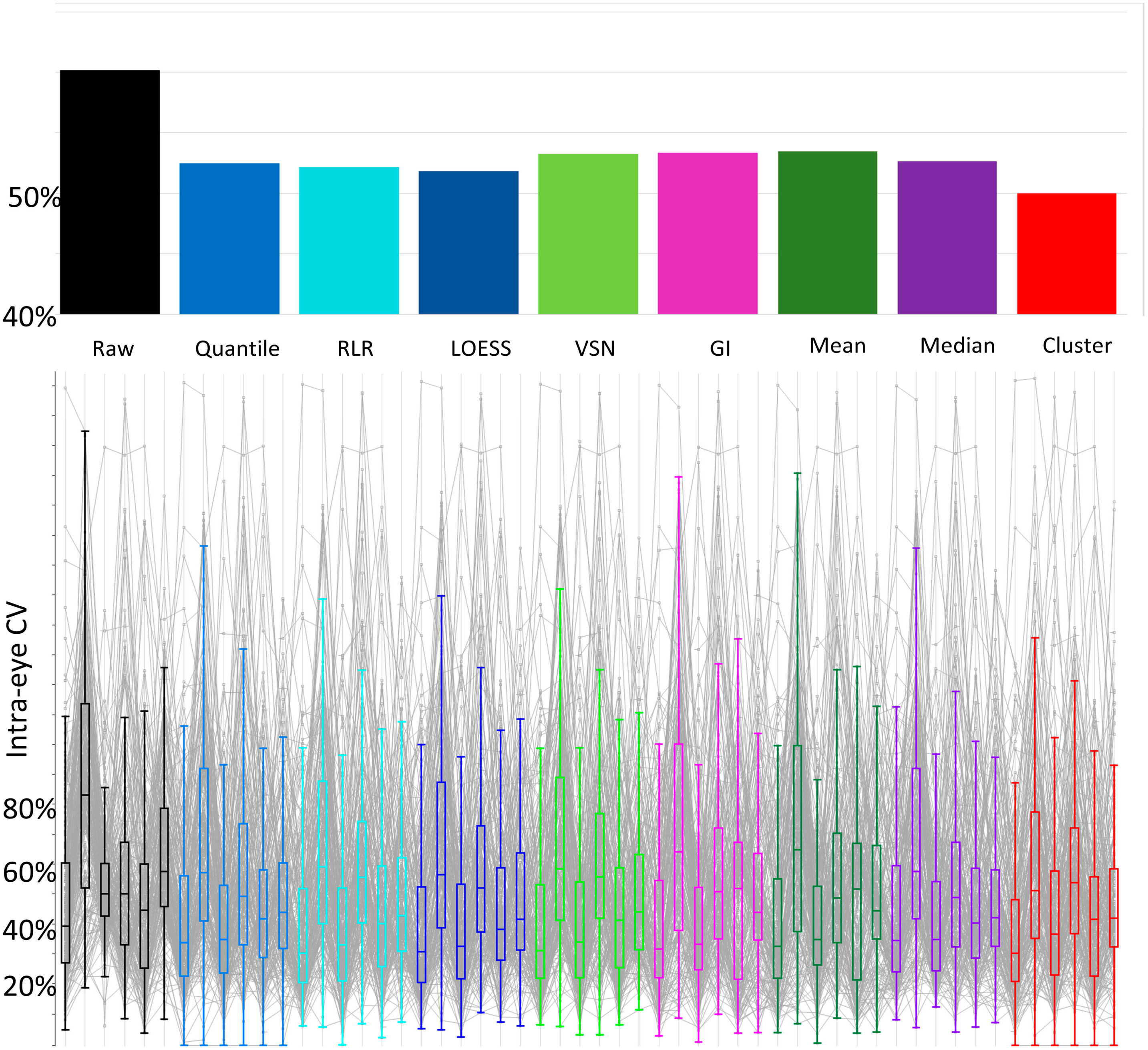

Figure S5. Intra-eye variance distribution of measured protein intensities after different normalization methods. Figure shows distribution of variances in each studied eye, median values of all proteins in all eyes are shown on top.

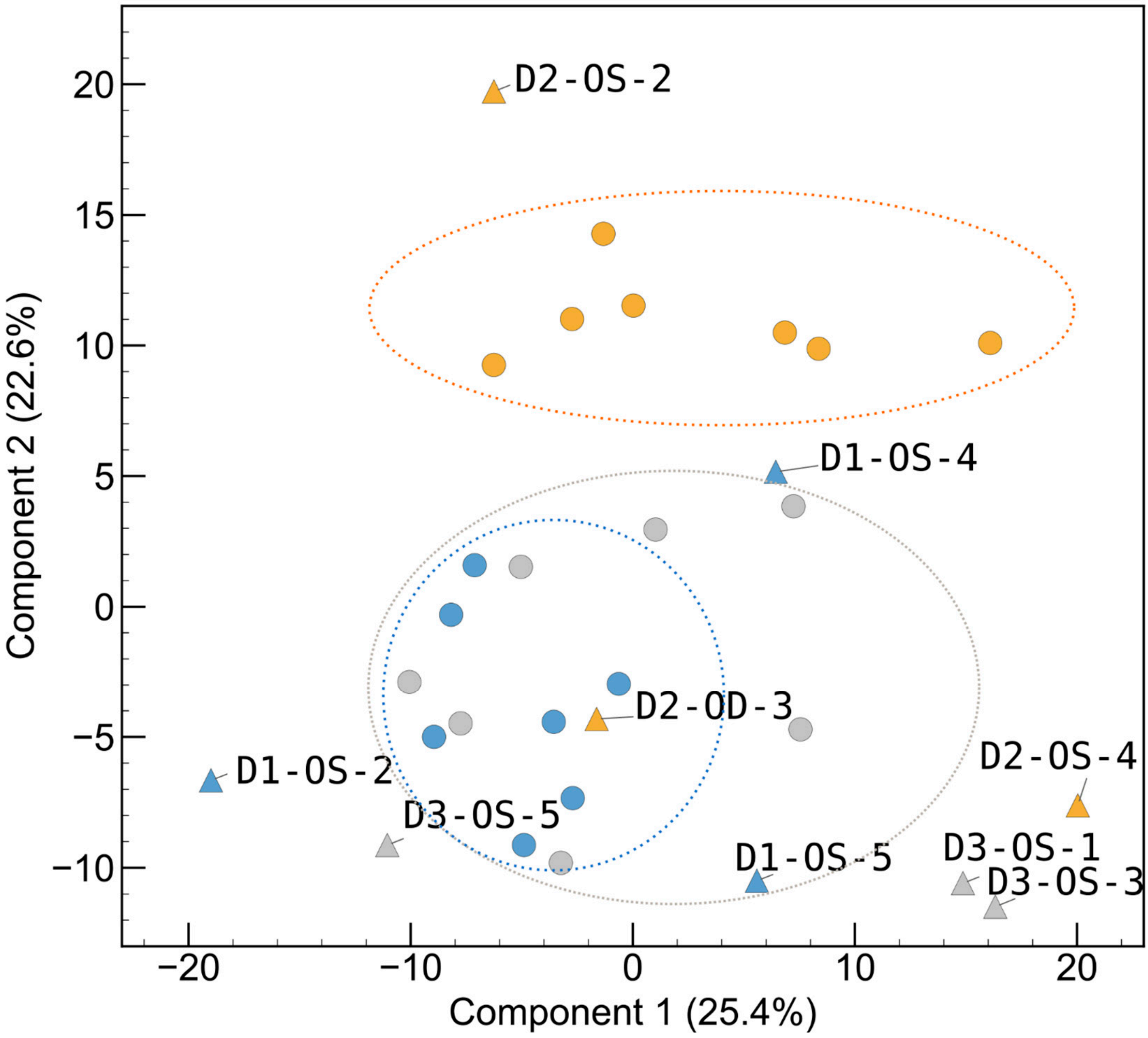

Figure S6. Identification of representative samples using Principal Component Analysis. Outlier samples are labeled with sample ID and shown with triangles.

Supplementary **Figure S7.**

Example MS/MS spectra of identified glycosylated peptides.

Diagnostic carbohydrate reporter ion peaks are coloured blue.

LACRT

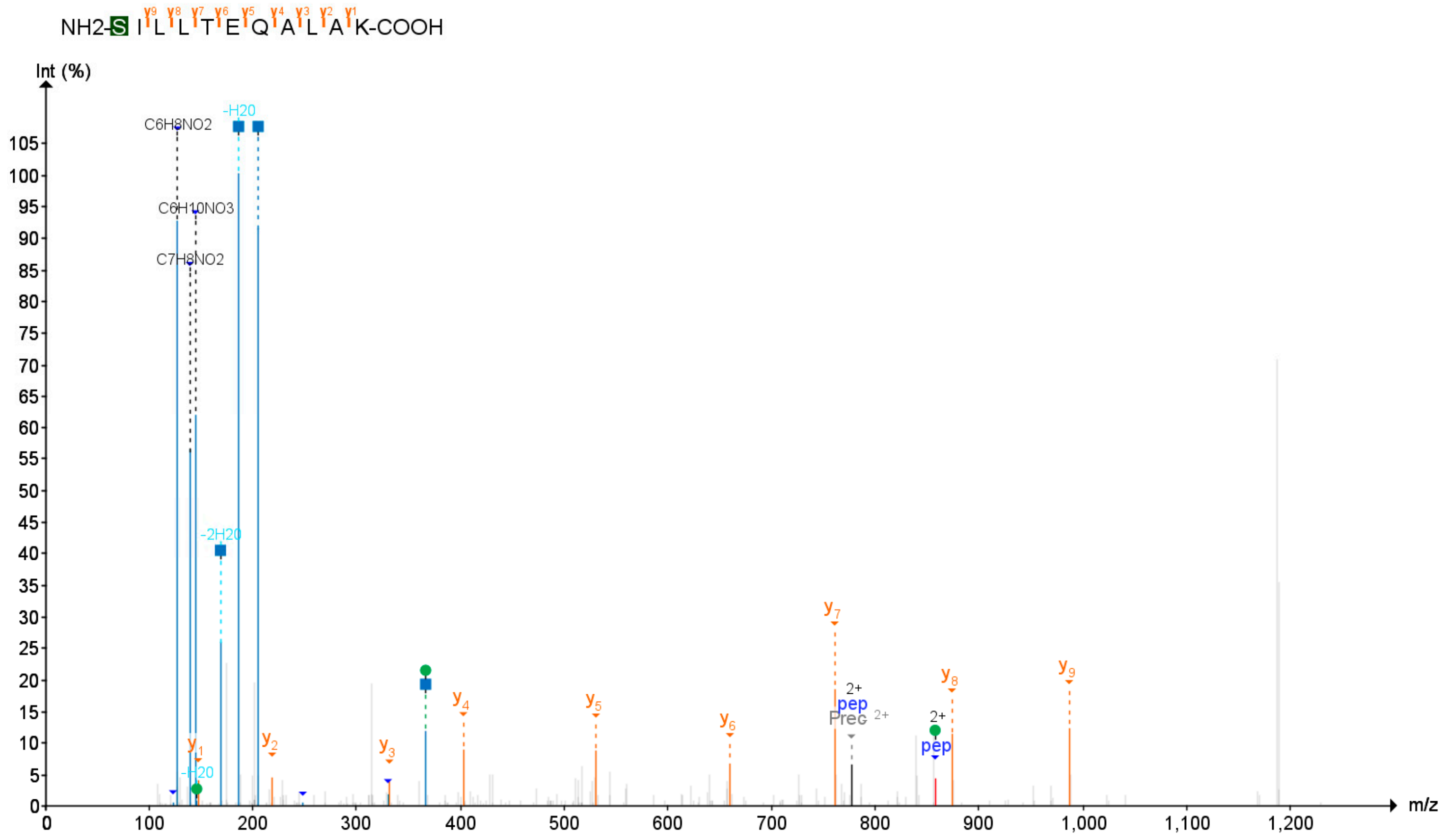

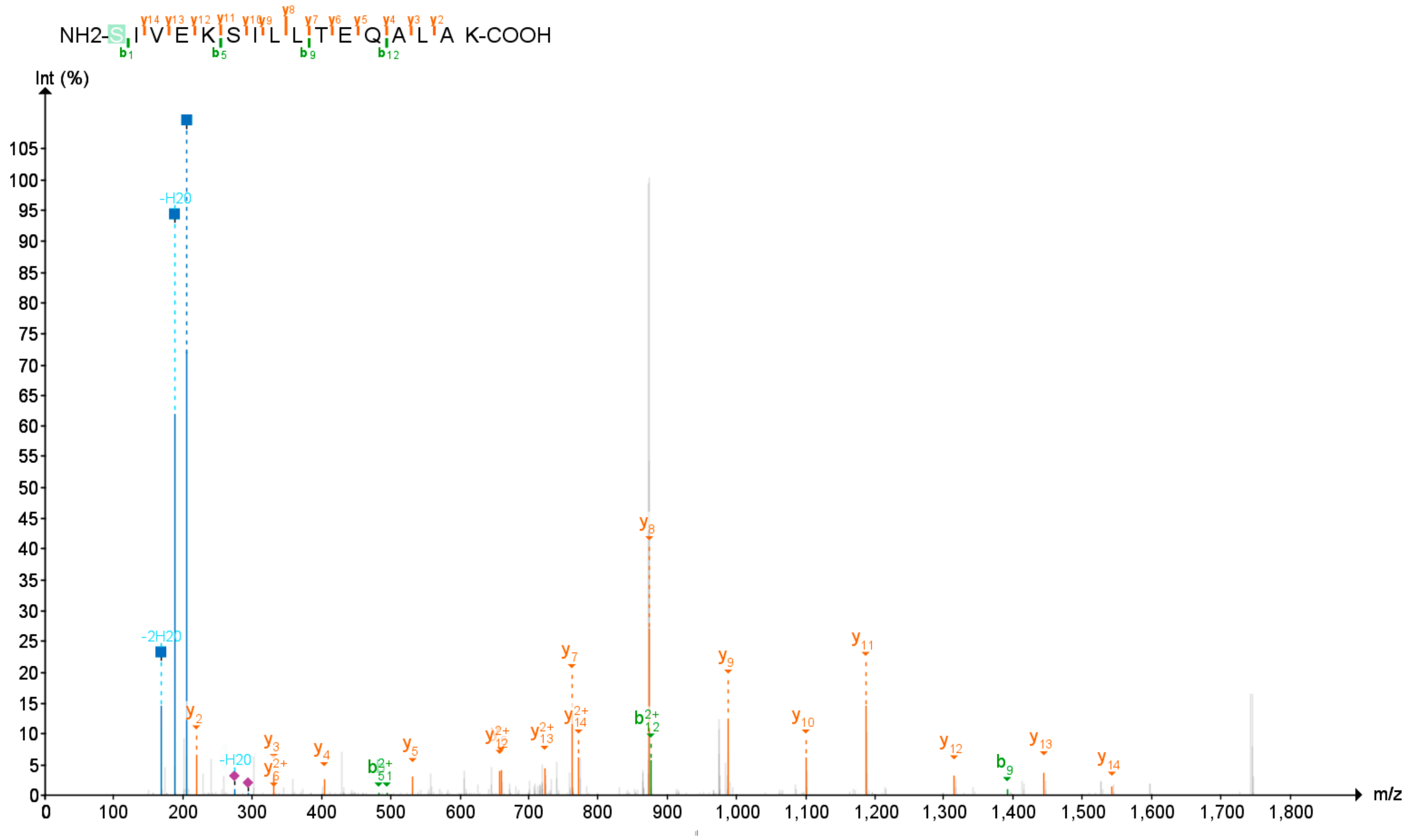

Supplement: Supplementary file 1 [file ijms-25-01559-s001.zip › Supplementary_Figures.pdf]
